# Time domain adaptation of left ventricular diastolic intraventricular pressure in elite female ice hockey athletes

**DOI:** 10.3389/fcvm.2023.1057129

**Published:** 2023-02-14

**Authors:** Ping Yang, Jianmei Zhang, Jun Xue, Yunfei Bai, Hui Yang, Ruiping Zhang, Benxiang He

**Affiliations:** ^1^Postdoctoral Mobile Station of Sports Science, Chengdu Sport University, Chengdu, Sichuan, China; ^2^Institute of Sports Medicine, General Administration of Sport of China, Beijing, China; ^3^National Emergency Medical Research Center, Emergency General Hospital, Beijing, China; ^4^Department of Physical Education, Jiangxi Science and Technology Normal University, Nanchang, Jiangxi, China

**Keywords:** athlete's heart, ice hockey, intraventricular pressure difference (IVPD), diastolic hemodynamics, vector flow mapping (VFM)

## Abstract

**Background:**

Ice hockey is a high-intensity dynamic sport for which competitive athletes train for longer than 20 hours each week for several years. The cumulative time of myocardial exposure to hemodynamic stress affects cardiac remodeling. However, the intracardiac pressure distribution of the elite ice hockey athletes' heart during adaptation to long-term training remains to be explored. This study aimed to compare the diastolic intraventricular pressure difference (IVPD) of the left ventricle (LV) between healthy volunteers and ice hockey athletes with different training times.

**Methods:**

Fifty-three female ice hockey athletes (27 elite and 26 casual) and 24 healthy controls were included. The diastolic IVPD of the LV during diastole was measured by vector flow mapping. The peak amplitude of the IVPD during isovolumic relaxation (P0), diastolic rapid filling (P1), and atrial systole (P4); the difference in the peak amplitude between adjacent phases (DiffP01, DiffP14); the time interval between the peak amplitude of adjacent phases (P0P1, P1P4); and the maximum decrease rate in diastolic IVPD were calculated. Differences between groups, as well as correlations between hemodynamic parameters and training time, were analyzed.

**Results:**

Structural parameters of the LV were significantly higher in elite athletes than in casual players and controls. No significant difference in the peak amplitude of the IVPD during the diastolic phase was found among the three groups. The analysis of covariance with heart rate as a covariate showed that P1P4 in the elite athlete and casual player groups was significantly longer than that in the healthy control group (*p* < 0.001 for all). An increased P1P4 was significantly associated with an increased training year (β = 4.90, *p* < 0.001).

**Conclusions:**

The diastolic cardiac hemodynamics of the LV in elite female ice hockey athletes could be characterized by a prolonged diastolic IVPD, and P1P4 prolonged with an increase in the training years, reflecting a time–domain adaptation in diastolic hemodynamics after long-term training.

## 1. Introduction

Long-term intense exercise induces adaptive changes in cardiac structure and function, which enable the heart to meet hemodynamic demands during exertion (1). The intraventricular pressure difference (IVPD) between the apex and the base of the left ventricle (LV) is the driving force that generates hemodynamics (2); however, the IVPD during adaptation to a prolonged training load remains to be explored.

Detailed study of diastolic hemodynamic adaptation to training load could provide new insights into the cardiac diastolic remodeling (3). A previous study showed that the peak reverse ejection IVPD between the apex of the LV and the outflow tract is useful to improve the assessment of relaxation of the LV using Doppler echocardiography (4). Since suction plays an important role in promoting early rapid filling and accepting an adequate filling volume under low filling pressure, the IVPD may be an effective marker to assess diastolic suction (5). Changes in the intraventricular pressure gradient (IVPG) suggest that the increase in suction and elastic recoil of the LV are directly correlated with improvements in LV relaxation (6). Using the IVPD to assess impaired diastolic suction, the results showed that patients with dilated cardiomyopathy have abnormally low diastolic suction and a blunted capacity to recruit suction with stress (7). In addition, the peak untwisting rate of the LV strongly correlated with the IVPG during isovolumic relaxation (8). Therefore, the IVPD between the apex and the base of the LV is an intuitive index to evaluate the diastolic hemodynamic characteristic of the LV.

The myocardial adaptation to exercise is expected to result from the exercise intensity and the amount of time during which hemodynamic stress acts on the cardiovascular system (9). With regard to exercise intensity, the Mitchell criteria are used to categorize sports according to the relative amount of static and dynamic skeletal muscle use and the cardiovascular effect of exercise (9, 10). In moderate-to-high-intensity dynamic exercise, volume and pressure load are imposed on the myocardium, and are presumably the greatest stimuli for structural and functional adaptation (11). Ice hockey is a high-intensity dynamic and medium-intensity static combination of exercise, requiring high endurance and explosive power. Female ice hockey players require high levels of both aerobic and anaerobic capabilities, along with strength, power, and agility (12). In this regard, the cardiovascular system has to increase its workload compared with moderate-intensity exercise (13), which potentially leads to adverse cardiovascular system adaptations (14). A previous study showed that training for competitive ice hockey induces LV cavity dilation and increased LV mass, and the associated cardiac adaptations serve to normalize wall stress (15). However, the IVPD in the dilated LV cavity with an increased LV mass in female ice hockey athletes remains to be explored.

Researchers have postulated that repeated insults to the ventricle following prolonged and intensive endurance exercise (probably >20 h/week for >20 years) may lead to irreversible ventricular remodeling (13). Transthoracic echocardiographic changes are strongly related to the training years and the weekly training time (WTT), and these reflect physiological adaptation of the heart to physical exertion in high-endurance athletes (16). Competitive athletes train for 20–40 h each week and push the physiological limits of the human body (17). Therefore, studying the diastolic IVPD characteristics in female ice hockey athletes with different training years/WTT could help to clarify the diastolic hemodynamic characteristics of athletes adapted to long-term training.

Relative pressure imaging enables the non-invasive quantification of the IVPD using vector flow mapping (VFM) and visualization of the regional pressure distribution (18). VFM measures the pressure difference from the apex of the LV to the base, and it has been used to detect myocardial dysfunction in coronary heart disease, hypertension, diabetes mellitus, end-stage renal disease, and aortic valve regurgitation (19, 20). Therefore, the IVPD based on VFM appears to be a reliable index to quantify the hemodynamics of the LV. This study aimed to examine the diastolic IVPD characteristics of the LV in female ice hockey athletes with different training times. Based on previous studies about the athletes' heart could be characterized by higher ratio of early to late atrial mitral Doppler peak flow velocity, longer isovolumic relaxation and deceleration times (21), and increased untwist rate (22), which can aid diastolic function; we hypothesized that female ice hockey athletes would demonstrate higher amplitude and longer duration of diastolic IVPD of the LV compared with healthy control, and that correlations would exist between hemodynamic parameters and training time.

## 2. Materials and methods

### 2.1. Participants

This study was performed in accordance with the Helsinki Declaration, and was approved by the Ethics Committee of The Institute of Sports Medicine of the General Administration of Sport of China (2021-15). All of the participants provided written informed consent. The participants also volunteered to participate in a cardiac function assessment study from April 2021 to January 2022 during the period of medical supervision and physical examination. All of the examinations were performed during the in-competition phase and 24 h following the last athletic training. Detailed medical history and training regimens were obtained along with routine physical examinations. The basic information of the participants, such as the age, training years, athlete's technical hierarchy, WTT in the last 6 months, height, weight, body surface area, heart rate, systolic blood pressure, and diastolic blood pressure, was collected.

The inclusion criteria were as follows: (1) women aged 18–30 years; (2) athletes who played ice hockey and healthy controls without regular exercise habits; (3) no specific medical history or chronic illness; and (4) normal cardiac structure and sinus rhythm. The exclusion criteria were as follows: (1) poor echocardiographic image quality, and (2) abnormal segmental ventricular wall movement. Seventy-seven women (53 athletes and 24 non-athletes) were included in the study.

We referred to the ice hockey athlete's technical hierarchy standards promoted by the Competitive Sports Department of the General Administration of Sport of China (23). The technical hierarchy of national team athletes who participated in world class championships was set to 4; who came in the top three in national competitions held by the General Administration of Sport of China was set to 3; who participated in national championships was set to 2; who participated in provincial championships was set to 1, and who were non-athletes was set to 0. Twenty-seven ice hockey athletes with a technical hierarchy of 3 or 4 were assigned to the elite athlete group, with a mean training year of 15 yrs and mean WTT of 24 h. Twenty-six ice hockey athletes with a technical hierarchy of 1 or 2 were assigned to the casual player group, with a mean training year of 5.5 yrs and mean WTT of 4.5 h. Twenty-four healthy women with irregular exercise habits were assigned to the healthy control group.

### 2.2. Transthoracic echocardiography

Using the approach recommended by the American Society of Echocardiography (24), a standard examination was performed using the LISENDO880 ultrasound system equipped with a phased array probe (S-121) with a scanning frequency range of 1.0–5.0 MHz (Aloka, Hitachi Tokyo, Japan). Two-dimensional imaging of the LV was performed to obtain the best possible images of the endocardium without foreshortening of the cavity of the LV or echo dropout. The Nyquist limit was set high enough to minimize aliasing. A frame rate of 60 Hz could provide a reasonably accurate estimation of relative pressure in three-dimensional flow from two-dimensional vector fields (25). Images were recorded with a frame rate of 60 Hz.

Echocardiographic measurements included the interventricular septal thickness, LV end-diastolic dimension, LV posterior wall thickness, LV end-systolic dimension, LV end-diastolic volume, LV end-systolic volume, peak early diastolic transmitral flow velocity (E peak), peak late diastolic transmitral flow velocity (A peak), ratio of early to late atrial mitral Doppler peak flow velocity (E/A), and cardiac output. The relative wall thickness was calculated as follows: (*interventricular septal thickness* + *LV posterior wall thickness*)/*LV end-diastolic dimension*. The LV mass (g) was calculated as: 0.8 × 10.4 × [(*interventricular septal thickness* + *LV posterior wall thickness* + *LV end-diastolic dimension*)^3^ – *LVend*−*diastolicdimension*^3^] + 0.6. The LV mass, LV end-diastolic volume, and LV end-systolic volume, as well as cardiac output were indexed to body surface area.

### 2.3. VFM analysis

**Figure 1:**
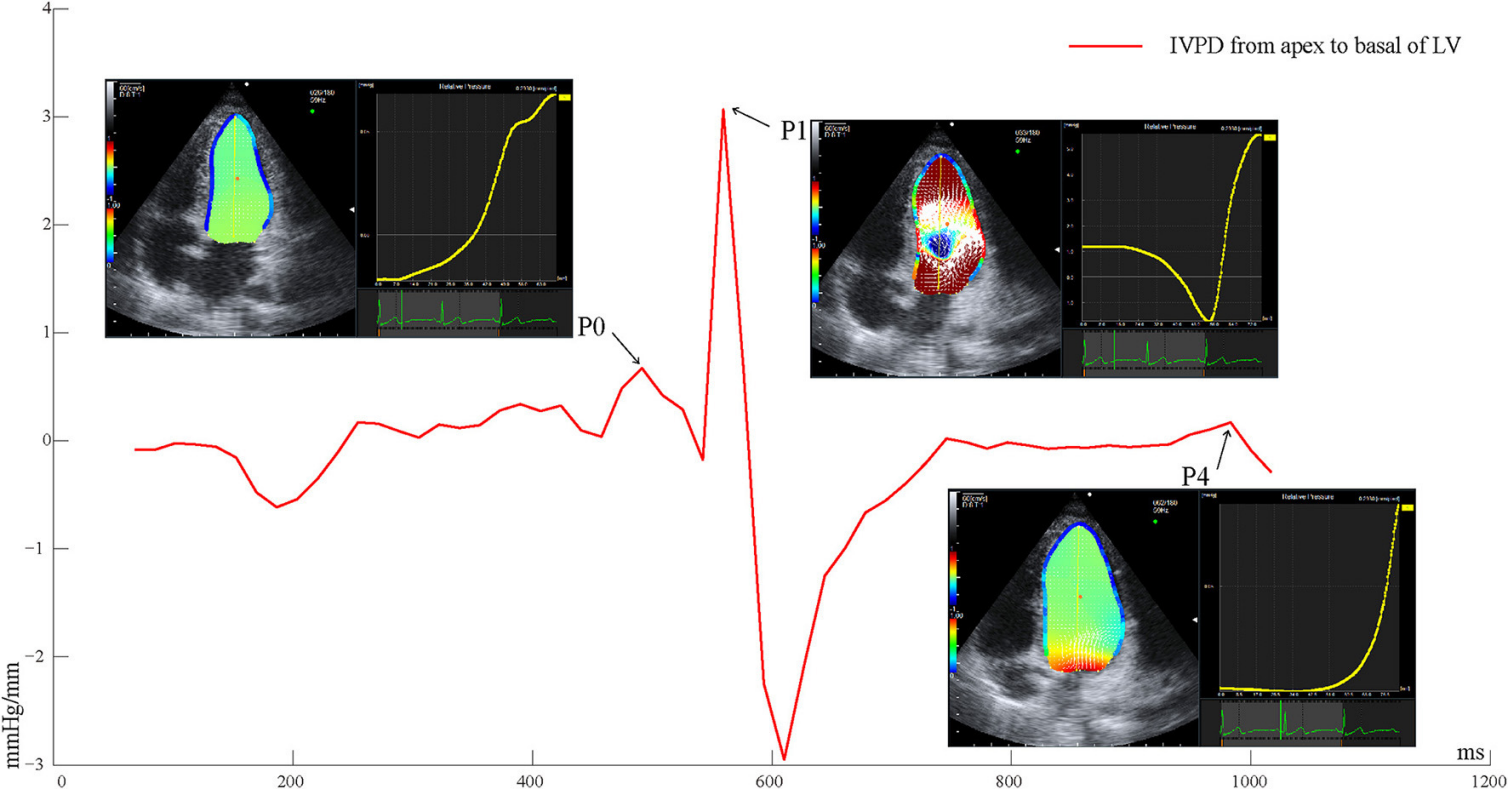
Time-IVPD curves of LV apical to basal of one cardiac cycle. LV, left ventricular; IVPD, intraventricular pressure difference; P0, isovolumic relaxation; P1, rapid filling; P4, atrial systole.

Echocardiographic images were captured over three successive beats, and color Doppler images in the apical four-chamber view were analyzed on the machine under the VFM configuration. Intraventricular flow velocity vectors of VFM were computed by solving the continuity equation using the flow velocity measurements obtained from color Doppler and the wall velocity measurements obtained from speckle-tracking echocardiography (5). The relative pressure imaging method was used to convert the velocity information into the relative pressure distribution using the momentum equations of fluid motion (Navier–Stokes equations) based on the VFM technology (Aloka, Hitachi Tokyo, Japan) (18). The endocardial border was manually traced at isovolumic relaxation and automatically determined throughout the remaining frames. By setting a sampling line from the apex to the base of the LV at isovolumic relaxation, a pressure difference between both ends of the line was calculated automatically for the whole cardiac cycle. The data of three cardiac cycles of the IVPD from the apex to the base of the LV were exported. The frame number of the peak value during isovolumic relaxation (P0), rapid filling (P1), and atrial systole (P4) was uniquely identified by simultaneously satisfying the following three conditions: (1) Valve opening and closing (P0 mitral and aortic valves closed; P1 and P4 mitral valve open, aortic valve closed), (2) Blood flow and direction (P0 blood flow was positively small and relatively static; P1 blood flow reached the first positive peak from the mitral valve to the apical direction; P4 had a positively small blood flow, and the blood flow was directed from the mitral valve to the apex of the LV), (3) The time-IVPD amplitude curve (as shown in Figure 1; P0 was the first small positive peak; P1 was the maximum positive peak value of the whole cardiac cycle; and P4 was the last positive peak during the diastolic period).

The peak values of the IVPD and IVPG (calculated by the IVPD divided by the length of the sample line) were extracted referring to the frame numbers of P0, P1, and P4. Differences in the peak IVPD between P0 and P1 (DiffP01), and between P1 and P4 (DiffP14) were calculated. The time intervals between P0 and P1 (P0P1), and between P1 and P4 (P1P4), were calculated by examining the frame differences between the peak IVPD of P0 and P1, and between the peak IVPD of P1 and P4, divided by the frame frequency. The peak rate of pressure decline was calculated through the steepest downward (negative) slope of the time–IVPD curve, reflecting the maximal decay in chamber pressure as the LV relaxed. All of the parameters were averaged over three consecutive cardiac cycles. The offline analyses of the IVPD parameters were processed with Matlab (MathWorks Inc., Natick, MA, USA).

### 2.4. Statistical analysis

All continuous variables were tested for normality using the Shapiro-Wilk test. If the data did not conform to a normal distribution, the transformation process was performed. Variables with a normal distribution are expressed as the mean ± standard deviation. Non-parametric variables are presented as the median [25th to 75th percentile]. Differences in continuous parameters were compared using one-way analysis of variance or the non-parametric Kruskal–Wallis test, as applicable, while the Bonferroni test was used to adjust the α level for pairwise comparisons. To control for the effect of heart rate on hemodynamic parameters, an analysis of covariance among groups of hemodynamic parameters (P0P1 and P1P4) was performed with heart rate as a covariate. Correlation analyses were used to analyze the relationships between the hemodynamic parameters and training time, basic physiological information, and echocardiography data. A multiple linear regression analyses for P1P4 and training time, physiological parameters, LV structural, and functional parameters, which was significantly correlated, was also conducted. Statistical significance was set at a *p* value of < 0.05. All calculations were performed using IBM SPSS 20 (IBM Corp. Armonk, NY, USA).

## 3. Results

### 3.1. Participants' basic characteristics

There was no significant difference in age among the three groups. Elite athletes had a higher technical hierarchy (4 [4 to 4] vs. 2 [0 to 3] grade, *p* < 0.001), a longer training years (15 [11 to 18] vs. 5.5 [2 to 8] years, *p* = 0.001) and a longer WTT (24 [24 to 24] vs. 4.5 [3 to 6] h, *p* < 0.001) than causal players (Table 1). The height (168 [165.5 to 170] vs. 161.50 [160 to 167.5] cm, *p* = 0.005), weight (63.58 ± 6.97 vs. 56.54 ± 8.78 kg, *p* = 0.003), and body surface area (1.72 ± 0.10 vs. 1.61 ± 0.13 kg/m^2^, *p* = 0.003) in the elite athlete group were significantly higher than in the healthy control group (Table 1). The heart rate in the elite athlete and casual player groups was significantly lower than in the healthy control group (67.41 ± 8.18 vs. 67.23 ± 10.62 vs. 76.75 ± 10.89 bpm, *p* = 0.007 for elite athletes vs. healthy controls, *p* = 0.004 for casual players vs. healthy controls; Table 1). The diastolic blood pressure in the elite athlete group was significantly lower than in the healthy control group (66 [64 to 71] vs. 74 [70 to 80] mmHg, *p* = 0.002; Table 1).

**Table 1:** Basic information of participants.

	**Elite athlete (*n =* 27)**	**Casual player (*n =* 26)**	**Healthy control (*n =* 24)**
Age, yrs	23.89 ± 3.50	22.81 ± 3.01	23.96 ± 2.37
Technical hierarchy	4 [4 to 4]^**††^	2 [0 to 3]^§^	0
Training year, yrs	15 [11 to 18]^**††^	5.5 [2 to 8]^§§^	0
Weekly training time, h	24 [24 to 24]^**††^	4.5 [3 to 6]^§§^	0
Height, cm	168 [165.5 to 170]^**^	165 [162 to 171]	161.50 [160 to 167.5]
Weight, kg	63.58 ± 6.97^**^	58.85 ± 5.89	56.54 ± 8.78
Body surface area, kg/m^2^	1.72 ± 0.10^**^	1.66 ± 0.10	1.61 ± 0.13
Heart rate, bpm	67.41 ± 8.18^**^	67.23 ± 10.62^§§^	76.75 ± 10.89
Systolic blood pressure, mmhg	113 [108 to 116.5]	110 [108 to 110]	110 [110 to 116.5]
Diastolic blood pressure, mmhg	66 [64 to 71]^**^	70 [68 to 80]	74 [70 to 80]

Values are presented as the mean ± standard deviation or median [25^th^ to 75^th^ percentile]. ^**^The difference between elite athletes and healthy controls was extremely significant after adjustment; ^††^The difference between elite athletes and casual players was extremely significant after adjustment; ^§, §§^The difference between casual players and healthy controls was significant and extremely significant respectively after adjustment. *p* < 0.05 for significant difference, *p* < 0.01 for extremely significant difference.

### 3.2. Echocardiographic parameters

The interventricular septal thickness (9.4 [8.3 to 10] vs. 8 [7 to 8] vs. 8 [8 to 8.65] mm, *p* = 0.002 for elite athletes vs. casual players, *p* < 0.001 for elite athletes vs. healthy controls) in the elite athlete group was significantly higher than in the casual player and healthy control groups (Table 2). The LV posterior wall thickness in the elite athlete group was significantly higher than in the healthy control group (8.3 [8.3 to 9.35] vs. 8 [8 to 9] mm, *p* = 0.01; Table 2). LV end-diastolic dimension (48.30 ± 2.99 vs. 43.84 ± 3.52 vs. 42.47 ± 4.08 mm, *p* < 0.001 for all), LV end-systolic dimension (29.4 [28.15 to 31.4] vs. 27.5 [25 to 29.4] vs. 25 [24 to 26] mm, *p* = 0.049 for elite athletes vs. casual players, *p* < 0.001 for elite athletes vs. healthy controls) in the elite athlete group were significantly higher than in the casual player and healthy control groups (Table 2).

**Table 2:** Echocardiographic parameters in elite athletes, casual players, and healthy controls.

	**Elite athlete** **(*n =* 27)**	**Casual player** **(*n =* 26)**	**Healthy control** **(*n =* 24)**
Interventricular septal thickness, mm	9.4 [8.3 to 10]^**††^	8 [7 to 8]	8 [8 to 8.65]
LV end-diastolic dimension, mm	48.30 ± 2.99^**††^	43.84 ± 3.52	42.47 ± 4.08
LV posterior wall thickness, mm	8.3 [8.3 to 9.35]^†^	8 [7.8 to 8]	8 [8 to 9]
LV end-systolic dimension, mm	29.4 [28.15 to 31.4]^**†^	27.5 [25 to 29.4]^§^	25 [24 to 26]
Relative wall thick, ratio	0.37 [0.36 to 0.39]	0.36 [0.33 to 0.39]	0.39 [0.36 to 0.41]
LV mass index, g/m^2^	82.27 [77.32 to 95.50]^**††^	66.86 [56.48 to 76.09]	67.76 [56.72 to 75.54]
LV end-diastolic volume index, mL/m^2^	63.91 ± 9.56^**††^	52.96 ± 9.29	49.85 ± 10.69
LV end-systolic volume index, mL/m^2^	19.63 [17.86 to 22.36]^**^	17.73 [14.48 to 21.35]^§§^	13.81 [12.71 to 14.78]
Sample line from LV apex to base, mm	81.66 ± 6.43	79.81 ± 7.92	77.63 ± 7.61
E peak, cm/sec	89.37 ± 15.07	83.29 ± 14.87	84.17 ± 15.12
A peak, cm/sec	48.77 ± 11.23	42.22 ± 8.34^§§^	53.17 ± 11.87
E/A, ratio	1.81 [1.71 to 2.01]	1.96 [1.71 to 2.14]^§§^	1.50 [1.38 to 1.96]
Cardiac index, L/min/m^2^	2.96 [2.72 to 3.22]^†^	2.28 [1.97 to 2.88]	2.78 [2.5 to 3.13]

Values are presented as the mean ± standard deviation or median [25^th^ to 75^th^ percentile]. ^**^The difference between elite athletes and healthy controls was extremely significant after adjustment;^†^,^†^^†^The difference between elite athletes and casual players was significant and extremely significant respectively after adjustment; ^§, §§^The difference between casual players and healthy controls was significant and extremely significant respectively after adjustment; *p* < 0.05 for significant difference, *p* < 0.01 for extremely significant difference.

The LV mass index (82.27 [77.32 to 95.50] vs. 66.86 [56.48 to 76.09] vs. 67.76 [56.72 to 75.54] g/m^2^, *p* < 0.001 for all) in the elite athlete group was significantly higher than in the casual player and healthy control groups (Table 2). The LV end-diastolic volume index in the elite athlete group was significantly higher than in the casual player and healthy control groups (63.91 ± 9.56 vs. 52.96 ± 9.29 vs. 49.85 ± 10.69 mL/m^2^, *p* < 0.001 for all; Table 2). The LV end-systolic volume index in the elite athlete and casual player groups was significantly higher than in the healthy control group (19.63 [17.86 to 22.36] vs. 17.73 [14.48 to 21.35] vs. 13.81 [12.71 to 14.78] mL/m^2^, *p* < 0.001 for elite athletes vs. healthy controls; *p* = 0.009 for casual players vs. healthy controls; Table 2).

The cardiac index (2.96 [2.72 to 3.22] vs. 2.28 [1.97 to 2.88] L/min/m^2^, *p* = 0.007) in the elite athlete group was significantly higher than in the casual player group (Table 2). The A peak in the casual player group was significantly lower than in the healthy control group (42.22 ± 8.34 vs. 53.17 ± 11.87 cm/sec, *p* = 0.002; Table 2). The E/A in the casual player group was significantly higher than in the healthy control group (1.96 [1.71 to 2.14] vs. 1.50 [0.38 to 1.96], *p* = 0.003; Table 2).

### 3.3. Diastolic IVPD parameters

**Figure 2:**
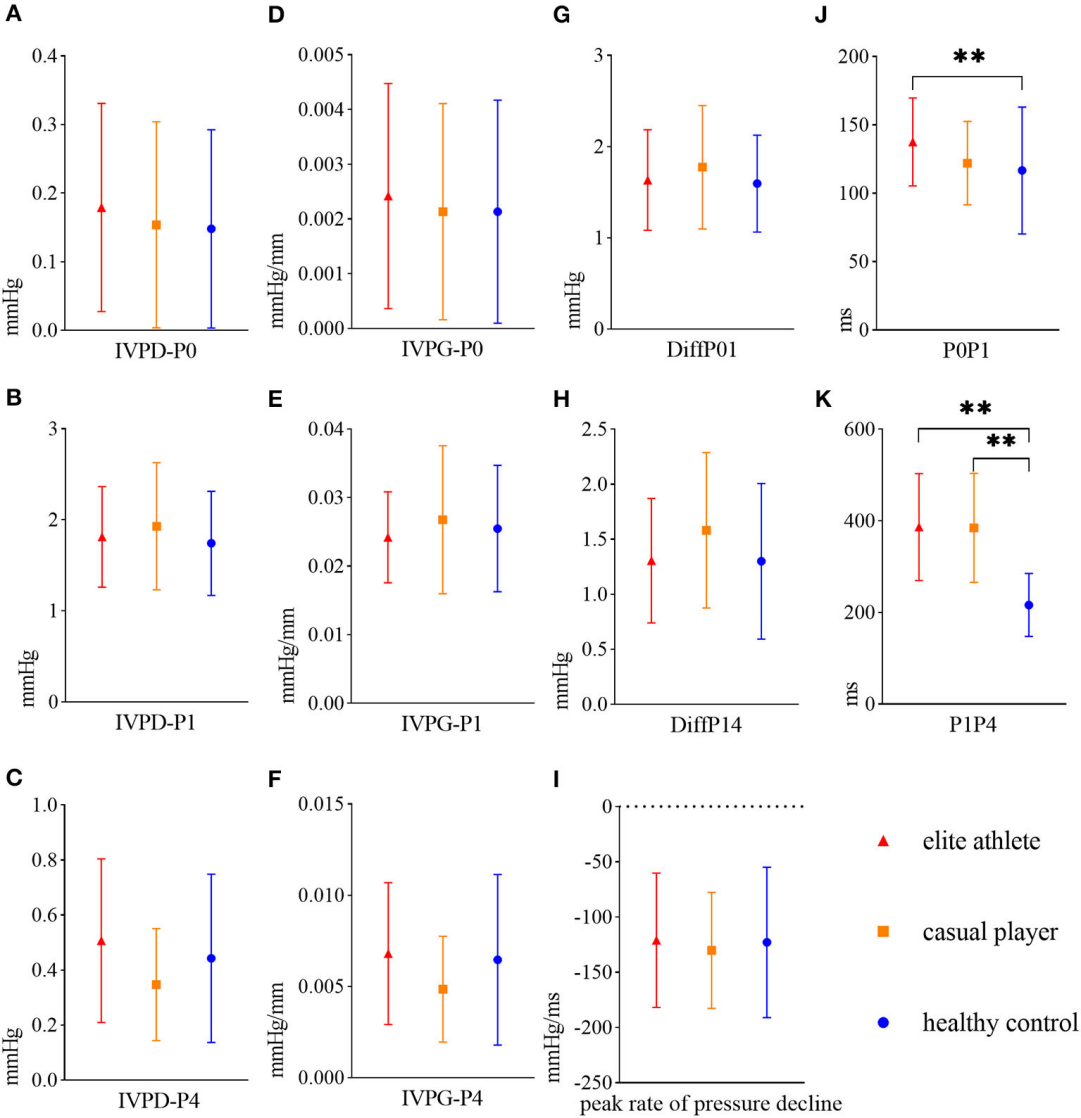
Mean and standard deviations of LV diastolic hemodynamic parameters in elite athletes (red triangles), casual players (orange squares), and healthy controls (blue circles). **(A)** Peak IVPD of P0 (IVPD-P0), **(B)** peak IVPD of P1 (IVPD-P1), **(C)** peak IVPD of P4 (IVPD-P4), **(D)** peak IVPG of P0 (IVPG-P0), **(E)** peak IVPG of P1 (IVPG-P1), **(F)** peak IVPG of P4 (IVPG-P4), **(G)** difference in the peak IVPD between P0 and P1 (DiffP01), **(H)** difference in the peak IVPD between P1 and P4 (DiffP14), **(I)** peak rate of pressure decline, **(J)** time interval between P0 and P1 (P0P1), **(K)** time interval between P1 and P4 (P1P4). **denotes *p* < 0.01. **(G–I)** has not been mentioned in the article.

There was no significant difference in the peak IVPD or IVPG in each diastolic phase among the groups (Figures 2A–F). P0P1 in the elite athlete group was significantly longer than in the healthy control group (136.09 ± 32.75 vs. 117.60 ± 47.87 ms, *p* = 0.046; Figure 2J). P1P4 in the elite athlete and casual player groups was significantly longer than in the healthy control group (388.14 ± 121.28 vs. 379.87 ± 118.69 vs. 211.41 ± 60.73 ms, *p* < 0.001 for all; Figure 2K). The analysis of covariance showed that P1P4 in the elite athlete and casual player groups was significantly longer than in the healthy control group (*p* < 0.001 for all), but the significant difference in P0P1 between the elite athlete group and the healthy control group disappeared.

### 3.4. Correlations of the WTT and P1P4 with basic physiological information, and echocardiographic and diastolic IVPD parameters

The WTT was significantly positively correlated with height, weight, body surface area, technical hierarchy, training year, interventricular septal thickness, LV end-diastolic/systolic dimension, LV mass index, sample line from LV apex to base, LV end-diastolic/systolic volume index, E/A, P0P1, and P1P4 (Table 3). Additionally, the WTT was significantly negatively correlated with heart rate and diastolic blood pressure (Table 3).

**Table 3:** Correlation of WTT and P1P4 with basic physiological information, echocardiographic and diastolic IVPD parameters.

	**WTT**		**P1P4**	
	**rho**	***p***	**rho**	***p***
Height	0.361	0.001^**^	0.137	0.235
Weight	0.420	<0.001^**^	0.230	0.004^*^
Body surface area	0.399	<0.001^**^	0.195	0.089
Technical hierarchy	0.935	<0.001^**^	0.456	<0.001^**^
Training year	0.910	<0.001^**^	0.491	<0.001^**^
WTT	1	NA	0.562	<0.001^**^
Heart rate	−0.344	0.002^**^	−0.742	<0.001^**^
Systolic blood pressure	0.016	0.889	−0.235	0.040^*^
Diastolic blood pressure	−0.414	<0.001^**^	−0.308	0.006^**^
Interventricular septal thickness	0.422	<0.001^**^	0.042	0.714
LV end-diastolic dimension	0.570	<0.001^**^	0.312	0.006^**^
LV end-systolic dimension	0.579	<0.001^**^	0.346	0.002^**^
Relative wall thick	−0.156	0.174	−0.276	0.015^*^
LV mass index	0.499	<0.001^**^	0.217	0.058
Sample line from LV apex to base	0.270	0.018^*^	0.133	0.250
LV end-diastolic volume index	0.479	<0.001^**^	0.295	0.009^**^
LV end-systolic volume index	0.528	<0.001^**^	0.369	0.001^**^
A peak	−0.176	0.125	−0.353	0.002^**^
E/A	0.270	0.018^*^	0.516	<0.001^**^
P0P1	0.299	0.008^**^	0.242	0.034^*^
P1P4	0.562	<0.001^**^	1	NA

LV, left ventricular; P0, isovolumic relaxation; P1, rapid filling; P4, atrial systole; rho, rank correlation coefficient of the non-parametric correlation analysis. ^*^denotes *p* < 0.05; ^**^denotes *p* < 0.01.

P1P4 was statistically significant after the covariance analysis, and thus the correlation and multiple linear regression analyses were conducted. P1P4 was significantly positively correlated with weight, technical hierarchy, training year, WTT, LV end-diastolic/systolic dimension, LV end-diastolic/systolic volume index, E/A, and P0P1 (Table 3). P1P4 was significantly negatively correlated with heart rate, systolic/diastolic blood pressure, relative wall thickness, and A peak (Table 3). The multiple linear regression analysis showed that an increase in P1P4 was related to an increase in the technical year (*p* < 0.001) and E/A (*p* < 0.001), as well as a decrease in heart rate (*p* < 0.001), systolic blood pressure (*p* = 0.007) and relative wall thickness (*p* = 0.009). The R^2^ value was 0.713 after model adjustment (*P1P4* = 1,127.37 – 6.21 × *heart rate* + 4.90 × *training year* + 104.48 × *E/A* – 3.08 × *systolic blood pressure* – 649.17 × *relative wall thickness*).

## 4. Discussion

The parameters of cardiac structure in elite athletes demonstrated significantly higher values than in casual players and healthy controls, which supported our hypothesis that female ice hockey athletes show cardiac structural remodeling. The peak amplitudes of the IVPD and IVPG in three diastolic phases were similar among groups, and thus these results did not confirm the hypothesis that female ice hockey athletes with different training year/WTT show differences in the cardiac diastolic IVPD of the LV. The main findings of the present study were that female elite ice hockey athletes (with more than 10 training years and WTT of longer than 20 h) had longer P1P4 than casual players (with fewer than 10 training years and WTT of shorter than 20 h) and healthy controls. An increase in P1P4 was correlated with an increase in the training year, which supported the hypothesis of correlations between hemodynamic parameters and training time.

### 4.1. The amplitude of the diastolic IVPD was similar between female ice hockey athletes and healthy controls

The peak amplitudes of the IVPD and IVPG in three diastolic phases were not significantly different among the three groups (Figures 2A–F). A previous study using four-dimensional flow magnetic resonance imaging supported our results, which showed no significant difference in the longitudinal impulse during early diastolic filling between athletes and controls (2). This lack of a significant difference in hemodynamics between the groups in this study may be correlated with the elastic mechanism of cardiac structural proteins. This mechanism drives ventricular elastic diastole and provides a spring-like power in the initial stage of rapid filling, and thus the blood in the left atrium can flow into the LV (26). Changes in the length of structural proteins during cardiac diastole affect the magnitude of elastic resilience, and the IVPG is affected only when the myocardium is impaired (e.g., myocardial ischemia) (3). This finding suggested that although there were differences in the morphological structure between the groups, the mechanism of intracardiac hemodynamics accelerating blood flow in the intracardiac cavity was similar. In addition, the IVPD was positively correlated with the longitude of the LV (27). Thus, no significant difference existed in the length of the sample line from the apex to the base of the LV among groups (Table 2), resulting in no significant difference between the groups in the peak amplitude of the IVPD in each diastolic phase.

### 4.2. Time-domain adaptation of the diastolic IVPD in female ice hockey athletes

In this study, the P0P1 and P1P4 in the elite athlete were significantly longer than in the healthy control groups (Figures 2J, K). Lower heart rate has been proven to predict endurance elite athletic status (28), and is correlated with relative extending diastole (2). Our results verified previous speculation that sinus bradycardia is related to a prolonged diastolic duration, as well as the time between the peak of early diastolic transmitral flow velocity and the peak of late diastolic transmitral flow velocity (29).

Pavlik et al. (30) concluded that an efficient stroke volume is caused by a higher active relaxation ability and a longer diastolic period, providing a greater Frank–Starling effect (i.e., an increased contractile force is proportional to the extent of diastolic myocardial fiber stretching). This effect achieves a higher cardiac performance at lower heart rates. Relevant rodent models (rats) have shown that the LV relaxation time constant (Tau) measured by cardiac intubation is increased in physiologically hypertrophic hearts, but decreased in pathologically hypertrophic hearts (31). Notably, in a previous study, the higher active relaxation ability and longer diastolic period correlated with the kinetic energy of blood flow in the LV (32). Combining this with the relationship of diastolic kinetic energy and LV vortex (33), we speculated that in the physiologically hypertrophic elite athlete's heart, the longer the vortex lasts, the longer the expansion of fluid kinetic energy to pressure is delayed, thus optimizing the pressure gradient distribution in the LV. These results suggest that optimization of LV hemodynamics in elite athletes is reflected by the duration of diastolic IVPD.

The correlation analysis showed that P1P4 was significantly positively correlated with technical hierarchy, training year and WTT. The multiple linear regression analysis showed that an increase in P1P4 was related to an increase in training year. The hypothesis that the training time would be significantly correlated with the hemodynamics of the LV was verified. Beaudry speculated that the cumulative years of exercise exposure would influence the extent of cardiac remodeling (9). Compared with 60–90 min of moderate-to-high-intensity training each day, several hours of training had a greater effect on the cumulative duration of cardiac exposure to high hemodynamic pressure (9). These findings indicate that prolongation of the training time increases the interaction time between the myocardium and hemodynamics, and further promotes extension of the cardiac cycle. Radovits et al. (34) found that exercise-induced physiological hypertrophy of the myocardium is characterized by improved active relaxation, reflecting optimization of myocardial energy use. These findings are in line with higher myocardial compliance in endurance athletes, which allows the LV to easily dilate with a higher efficient output (35). Therefore, alterations in the training time have a substantial impact on the maintenance of myocardial elastic relaxation.

### 4.3. Cardiac structural remodeling in female ice hockey athletes

The cardiac structural parameters of the LV in elite athletes showed significantly higher than those in casual players and healthy controls (Table 2). These findings support our hypothesis that female ice hockey athletes show cardiac structural remodeling. Ice hockey is a high-intensity dynamic and medium-intensity static sport, but cardiac structure suggested adaptive changes in endurance training. Unlike pathological dilatation of the heart, the athlete's heart adapts to long-term endurance training, and its overall geometry and the proportion of each chamber remain unchanged (36). Slight dilatation of the structural index of the athlete's heart within the normal range results from adaptation to a regular training load (37). According to Morganroth, high-intensity endurance exercise increases venous return volume along with cardiometabolic demand (38). This situation increases ventricular preload and induces morphological changes in myocardial fibers through diastolic stress.

The LV mass index in elite athletes was significantly higher than in casual players and healthy controls (Table 2). This observation is consistent with a previous study showing that exercise-induced cardiac remodeling is related to an increase in the LV mass, which is correlated with an increase in venous return volume and cardiometabolic demand (39). Steding-Ehrenborg et al. (33) concluded that LV mass was an independent predictor of the diastolic kinetic energy of the LV. Athletes' hearts adapt to the large volume of blood pumping during exercise, blood flow organization is improved in the heart cavity through kinetic energy optimization, and 70% of the diastolic kinetic energy comes from the LV diastolic vortex (33). This suggests that a greater LV mass generates higher diastolic kinetic energy through greater diastolic vortex in elite athletes.

## 5. Limitations

This study has three main limitations. First, it is worth noting that the sampling line from LV apex to base was determined during the isovolumic diastolic phase (P0), and the pressure distribution of the sample line during other diastolic phases was automatically tracked with a fixed length of the sample line. However, the variation in the apex to base length may differed among the three groups. Therefore, the relative pressure distribution from the LV apex to the mitral valve with variations in the length of the sample line in LV diastole needs to be further explored. Second, as the adaptations in the athlete's heart mainly occur during exertion, future studies should focus on the IVPD during exertion in elite athletes. Third, sex differences exist in cardiac structural remodeling and function in athletes, but this study only focused on female ice hockey players. Therefore, further studies on the cardiac hemodynamic characteristics of male athletes should be considered, and correlations between hemodynamic parameters and maximal oxygen uptake, training intensity, and performance should be further examined to explore the hemodynamic mechanisms of cardiac adaptation to the training load.

## 6. Conclusions

The hearts of elite female ice hockey athletes show typical dilated remodeling. The diastolic cardiac hemodynamics of the LV in female elite ice hockey athletes could be characterized by the longer duration of the diastolic IVPD. Moreover, P1P4 prolonged with training year, reflecting a time–domain adaptation in cardiac diastolic hemodynamics in elite athletes. The results of this study provide an effective basis for understanding the hemodynamic mechanism of cardiac adaptation to long-term training and provide a reliable reference for the scientific training of female ice hockey athletes.

## Data availability statement

The raw data supporting the conclusions of this article will be made available by the authors, without undue reservation.

## Ethics statement

The studies involving human participants were reviewed and approved by Ethics Committee of the Institute of Sports Medicine of the General Administration of Sport of China. The patients/participants provided their written informed consent to participate in this study. Written informed consent was obtained from the individual(s) for the publication of any potentially identifiable images or data included in this article.

## Author contributions

PY and JZ (Equal & First) contributed to the conception and design of the study. JX and YB contributed to the acquisition of data. PY, HY, and RZ contributed to the analysis and interpretation of data. PY drafting the article and revising it critically for important intellectual content. BH final approval of the version to be submitted. All authors contributed to the article and approved the submitted version.
